# *PGK1* facilities cisplatin chemoresistance by triggering *HSP90/ERK* pathway mediated DNA repair and methylation in endometrial endometrioid adenocarcinoma

**DOI:** 10.1186/s10020-019-0079-0

**Published:** 2019-03-29

**Authors:** Jing-Wei Zhou, Juan-Juan Tang, Wei Sun, Hui Wang

**Affiliations:** Department of Gynecology, Jiangsu Province Hospital, Room 1711, No.220, Jiangdongbei Road, Gulou District, Nanjing, 210000 Jiangsu Province People’s Republic of China

**Keywords:** Chemoresistance, DNA methylation and repair, Endometrial carcinoma, ERK, HSP90

## Abstract

**Background:**

Endometrial carcinoma represents one of the most common cancer types of the female reproductive tract. If diagnosed at an early stage, the 5-year survival rate is promising. However, recurrence and chemoresistance remain problematic for at least 15% of the patients. In the present study, we aim to reveal the mechanism by which PGK1 regulates chemoresistance in endometrial carcinoma.

**Methods:**

qPCR was performed to detect expression of PGK1 in clinical tissue samples of endometrial carcinoma. Specific shRNAs were employed to knockdown PGK1 expression in endometrial cancer cell lines. MTT assay was used to evaluate cell viability and cisplatin sensitivity of endometrial carcinoma cell lines. Western blot was performed to assess the effects of PGK1 knockdown on the expression levels of HSP90, DNA repair-associated proteins (c-JUN, FOSL1, and POLD1), and DNA methylation-related enzymes (DNMT1, DNMT3A and DNMT3B). Immunoprecipitation was performed to verify direct binding between PGK1 and HSP90.

**Results:**

We first showed that PGK1 expression is elevated in tumor tissues of endometrial cancer, and high PGK1 levels are associated with clinical stages and metastasis. Knockdown of PGK1 inhibits proliferation of endometrial cancer cells, and enhances the inhibitory effect of cisplatin on cell viability. In addition, knockdown of PGK1 down-regulates the expression of DNA repair-related proteins, methylation-related enzymes, and total cellular methylation level. PGK1 was next shown to interact directly with HSP90 and exhibit pro-tumor effects by modulating the ATPase activity of HSP90.

**Conclusions:**

We propose that PGK1 mediates DNA repair and methylation through the HSP90/ERK pathway, and eventually enhances the chemoresistance to cisplatin. The results provide new insights on functions of PGK1 and HSP90, which might make them as promising targets for endometrial cancer chemotherapy.

## Background

Endometrial carcinoma, which commonly occurs after menopause, is a cancer originating from the endometrium (Morice et al., 2016; Wright et al., 2009). In developed countries, endometrial carcinoma represents the most common cancer type of the female reproductive tract. The endometrioid adenocarcinoma is the most common subtype, accounting for 80% of all endometrial carcinoma cases (Jemal et al., 2011). When diagnosed at an early stage without metastasis, the five-year survival rate is over 80% (Amant et al., 2005). Nevertheless, recurrence and resistance to chemotherapy, mediated by the up-regulation of efflux pump expression and mutations of β-tubulin, pose substantial challenges for 15–20% of the patients (Moxley & McMeekin, 2010). To date, the detailed molecular mechanisms for endometrial carcinoma malignancy and relapse remain elusive.

Phosphoglycerate kinase 1 (PGK1) is a key glycolytic enzyme that produces ATP by catalyzing the conversion of 1,3-diphosphoglycerate to 3-phosphoglycerate (Bernstein & Hol, 1998). In addition to its well-known functions in glycolysis, recent reports have confirmed the association of its abnormal expression with the poor prognosis in a variety of cancer types, including multidrug-resistant ovarian cancer (Duan et al., 2002), breast cancer (Zhang et al., 2005), radioresistant astrocytomas (Yan et al., 2012), colon cancer (Ahmad et al., 2013), gastric cancer (Zieker et al., 2010), and hepatocellular carcinoma (Ai et al., 2011). It is also recently reported that in endometrial cancer, PGK-1 expression is elevated and is statistically linked to a variety of pathological indicators (Guo et al., 2018). However, the specific roles and underlying mechanism of PGK1 in endometrial cancer remain largely unknown, and further exploration is required.

Heat shock protein 90 (HSP90) is a highly conserved molecular chaperone involved in cellular homeostasis and stress response (Taipale et al., 2010). Since a variety of client proteins serviced by Hsp90 have critical functions in cellular growth, Hsp90 has become a promising target for cancer therapy (Neckers, 2007). It has recently been shown in esophageal squamous cell carcinoma that receptor interacting protein kinase 3 (RIP3) regulates DNA repair through the HSP90/ERK pathway, and ultimately affects tumor chemoresistance (Sun et al., 2018). Additionally, it has been reported that terazosin activates HSP90 through PGK1, and thus enhances the tolerance of cellular stress (Chen et al., 2015). Moreover, it has been established that DNA methyltransferase 1 (DNMT1), which is responsible for DNA methylation, is also a downstream factor regulated by HSP90 in human pancreatic and colon cancers (Nagaraju et al., 2017).

In the present study, we revealed the roles of PGK1 and HSP90 in endometrial carcinoma. We found that PGK1 mediated DNA repair and methylation through regulating HSP90 activity, and eventually affected chemoresistance to cisplatin. The results provided essential information on the functions of PGK1 and HSP90 and establish them as potential targets for endometrial cancer chemotherapy.

## Methods

### Clinical sample collection

Clinical endometrial tumor tissues (*n* = 56) and normal human endometrial tissues (*n* = 20) were collected from People’s Hospital of Jiangsu Province. The clinicopathological characteristics of enrolled patients were show in Table 1. This study was approved by the Ethics Committee of People’s Hospital of Jiangsu Province, and an informed consent form was provided for all participants. All samples had confirmed pathological diagnosis and were assigned with stages according to the FIGO guidelines. The collected clinical tissue samples were washed with sterile phosphate-buffered saline (PBS), frozen with liquid N_2_, and stored at − 78 °C until use.

**Table 1:** Clinicopathological characteristics of enrolled endometrioid endometrial cancer patient

Clinicopathological data	No. Of patients
Age (years)	
< 62	25
≥ 62	31
Menopausal status	
Pre−/perimenopausal	29
Postmenopausal	27
FIGO stage	
I/II	19
III/IV	37
Histologic grade	
Grade 1	30
Grade 2/3	26
Myometrial invasion	
< 1/2	34
≥ 1/2	22
Metastatic lymph nodes	
Negative	14
Positive	42

### Cell culture

Human endometrial cancer cell lines Ishikawa and HEC1A were purchased from ATCC (USA). Cisplatin-resistant Ishikawa cell line (Ishikawa/DDP) was purchased from Fenghui Biotechnology (Hunan, China). HSP90 inhibitor 17-AAG was purchased from Cell Signaling Technology (8132S) and used at 1 μM. ERK inhibitor PD98059 was purchased from Cell Signaling Technology (9900S) and used at 20 μM. Cells were cultured in DMEM:F12 medium (Life Technologies, Carlsbad, USA) in a humidified incubator at 37 °C with 5% CO_2_. The medium was supplemented with 10% fetal bovine serum (FBS), 100 μg/ml streptomycin, and 100 U/ml penicillin. For treatments with cisplatin, the cells were placed in 96-well plates at 20–30% confluence and kept for 12 h before treatment.

### Cell transfection

shRNA sequence targeting PGK1 and overexpressing plasmid of PGK1 were purchased from GenePharma (Shanghai, China). Endometrial cancer cell lines Ishikawa and HEC1A were transfected with shRNA targeting PGK1 (shPGK1), negative control random scrambled shRNA (shNC), or empty vector for overexpression of PGK1 with the Lipofectamine 2000 transfection reagent (Invitrogen, Carlsbad, USA) following the manufacturer’s protocols. The modulated cells were applied to indicated experiments 48 h after the transfection.

### Total cell protein extraction and Western blot

Endometrial cancer cells were lysed with cold lysis buffer, and proteins were extracted followed by quantification of protein concentration. Equal amounts of proteins from each group were purified with 10% SDS-polyacrylamide gels, and then transferred to a polyvinylidene difluoride (PVDF) membrane, followed by blockading of non-specific binding by incubation with 5% skim milk over 1 h. The membrane was next incubated with primary antibodies overnight at 4 °C, and then treated with horseradish peroxidase-conjugated secondary antibody for 1 h. The ECL reagent (Millipore Corp.) was used for detecting antibody-reactive bands. The housekeeping proteins α-tubulin or β-actin was used as loading control. Primary antibodies against POLD1, JNK, p-JNK, c-JUN, and FOSL1 were purchased from Santa Cruz Biotech (Dallas, USA); primary antibodies against PGK1, HSP90, ERK, p-ERK, AKT, p-AKT, DNMT1, DNMT3A, DNMT3B, and SPARC were purchased from Cell Signaling Technology (Boston, USA); primary antibodies against Bcl-xL, Mcl-1, and Bax were purchased from Abcam (Cambridge, UK).

### Total RNA extraction and qPCR

Based on the manufacturer’s instructions, total cellular RNAs were extracted from endometrial tumor tissues with Trizol reagent (Invitrogen) and were reverse-transcribed to cDNA with the Prime-Script RT-PCR master mix (Takara). qRT-PCR detection of PGK1 expression level was performed with SYBR Green qPCR (Toyobo) according to the manufacturer’s protocols. GAPDH was used as an internal control. The primer sets used were as follow: PGK1 F: TCACTCGGGCTAAGCAGATT; PGK1 R: CAGTGCTCACATGGCTGACT; GAPDH F: CCAGGTGGTCTCCTCTGA; GAPDH R: GCTGTAGCCAAATCGTTGT.

### Detection of cellular ATP level

The cellular ATP levels were measured using the ENLITEN ATP Assay System (Promega, FF2000) based on the manufacturer’s instructions. Briefly, the cells were first washed and lysed in the ATP assay buffer. After addition of DMSO into the lysates, the total ATP level was detected immediately.

### Detection of cellular methylation level

Endometrial cancer cell lines Ishikawa and HEC1A were lysed with cold lysis buffer. The proteins were extracted from the lysates followed by quantification of protein concentration. Equal amounts of proteins from each group were purified with 10% SDS-polyacrylamide gels, and then transferred to a polyvinylidene difluoride (PVDF) membrane, followed by blockading of non-specific binding by incubation with 5% skim milk over 1 h. The membrane was next incubated with a 5-methylcytosine polyclonal antibody (ThermoFisher, PA1–30675, 1:1000) overnight at 4 °C, followed by treatment with horseradish peroxidase-conjugated secondary antibody for 1 h. The ECL reagent (Millipore Corp.) was used for detecting antibody-reactive bands.

### Co-immunoprecipitation

Hemagglutinin (HA)-tagged PKG1 (PKG1-HA) was expressed in Ishikawa cells. The cells were then lysed in a lysis buffer (20 mM Tris-HCl, pH 8.0, 10 mM KCl, 130 mM NaCl, 1.5 mM MgCl_2_, 10% glycerol) supplemented with protease inhibitors. After centrifugation at 4 °C for 10 min at 13,000 g, the supernatant was collected and incubated with anti-HA antibody (Signalway Antibody, College Park, USA) at 4 °C overnight. To precipitate the complexes, protein A/G-agarose beads were added. After thorough washing with lysis buffer, the beads were boiled in SDS loading buffer, followed by SDS-PAGE electrophoresis.

### MTT cell viability assay

Cell Proliferation Kit I (MTT, Roche Biotechnology) was used per the manufacturer’s instructions. 48 h after transfection with shRNA or overexpressing vector, the endometrial tumor cells (Ishikawa, HEC1A, or Ishikawa/DDP) were seeded in individual wells of a 96-well plate with 200 μL of culture medium and incubated for 24 h. For determination of IC_50_, the initial medium was removed and replaced with 100 μL of medium with a concentration gradient of cisplatin (1, 10, 20, 40, 60, 80, 100 μM for the parental cell line and 1, 50, 100, 200, 300, 400,500 for the cisplatin-resistant Ishikawa/DDP cell line) for 24 h. The IC_50_ values were calculated using GraphPad Prism 6. For drug combination effect assay, the initial medium was removed and replaced with 100 μL of medium containing cisplatin (20, 40, or 60 μM) and incubated for 72 h. 17-AAG or PD98059 were added 24 h before MTT labeling. Then, 10 μL of MTT labeling reagent was added to each well and incubated for 4 h. 100 μL of solubilization reagent was added followed by incubation overnight at 37 °C. Absorbance was measured at wavelengths of 550 nm and 690 nm by a microplate reader (Bio-Rad, USA).

### Flow cytometry cell apoptosis detection

The treated Ishikawa or HEC1A cells were collected and stained with the Annexin V-FITC apoptosis detection kit (APOAF, Sigma Aldrich), then washed twice with PBS followed by resuspension in 100 μL binding buffer supplemented with 5 μL annexin V-FITC. The sample was then stained with 5 μL propidium iodide (BD Biosciences, NJ, USA) for 10 min in dark, followed by addition of 400 μL of binding buffer. The resulting mixture was then analyzed with FACS Calibur. The Ishikawa or HEC1A cells stained with annexin V, propidium iodide, or both were designated as early apoptotic, necrotic, or late apoptotic, respectively. The data from flow cytometry were analyzed with BD FACSDiva (Becton-Dickinson, USA).

### Xenograft

BALB/c nude mice (6–8 weeks) were obtained from SLRC Experimental Animal (Shanghai, China). Prepared Ishikawa endometrial cancer cells (1 × 10^6^) transfected with sh-PGK1 or negative control shNC were injected subcutaneously into the right flank of mice. Tumor volume was measured and calculated every five days over 30 days to plot the tumor growth curve. The volume was calculated with the following formula: length x width^2^/2. The mice were euthanized in 30 days.

### Data analysis

GraphPad Prism 6.0 was used for statistical analysis. Student’s t-test was used to analyze the differences between two groups; one-way ANOVA and subsequent Tukey’s post hoc test were performed to determine significant differences among multiple groups. We considered *P* value lower than 0.05 (*P < 0.05) as statistically significant.

## Results

### PGK1 expression is elevated in endometrial cancer tissues and is associated with FIGO stages and metastasis

**Fig. 1:**
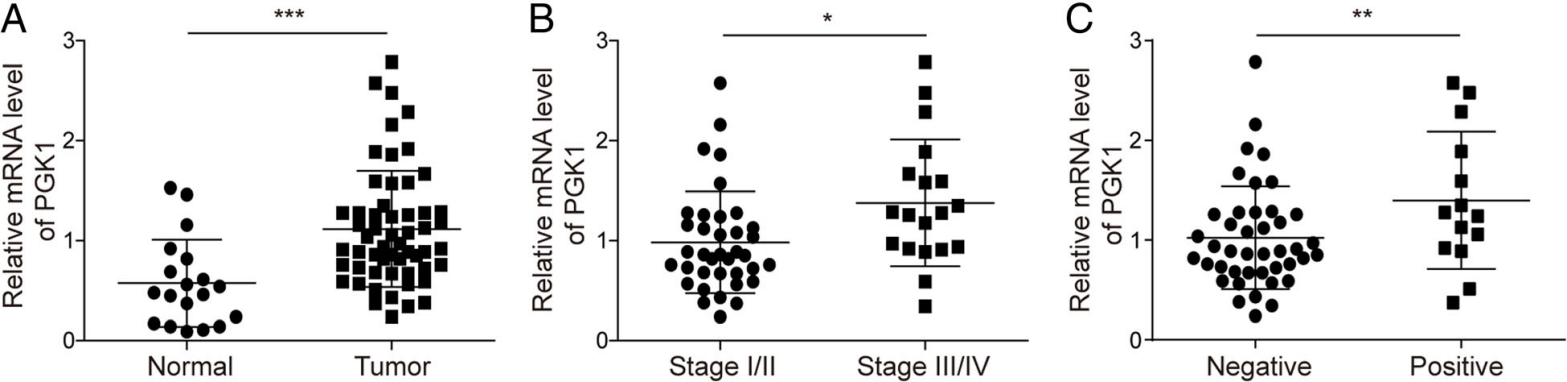
PGK1 expression is elevated in endometrial cancer tissues and is associated with FIGO stages and lymphatic metastasis. **a**. qPCR was used to detect the expression levels of PGK1 in endometrial carcinoma tissues (*n* = 56) and in normal human endometrial tissues (*n* = 20). **b**. qPCR was used to compare the expression levels of PGK1 in stage I/II endometrial carcinoma tissues (*n* = 37) and in stage III/IV endometrial carcinoma tissues (*n* = 19). **c**. qPCR was performed to examine the expression levels of PGK1 in endometrial carcinoma tissues with positive lymph node metastasis (*n* = 14) and with negative lymph node metastasis (*n* = 42). Data were shown as mean ± SD based on three independent experiments. ***, *P* < 0.001 compared with normal tissue; *, *P* < 0.05 compared with Stage I/II; **P < 0.01 compared with metastasis negative group

First, we evaluated the expression levels of PGK1 in clinical endometrial tumor tissues. Using qPCR, we observed markedly higher expression of PGK1 in endometrial tumor tissues (*n* = 56) compared with normal human endometrial tissues (*n* = 20, Fig. 1a). We next identified a correlation between the PGK1 expression level and the FIGO stages of the tumor tissues, and found higher expression level of PGK1 in stage III/IV endometrial tumor tissues (*n* = 37) than in stage I/II endometrial tumor tissues (*n* = 19, Fig. 1b). Furthermore, compared with the endometrial tumor tissues without lymph node metastasis (*n* = 42), elevated expression of PGK1 was observed in endometrial tumor tissues with lymph node metastasis (*n* = 14, Fig. 1c).

### PGK1 knockdown inhibits proliferation and enhances cisplatin sensitivity in endometrial cancer cell lines

**Fig. 2:**
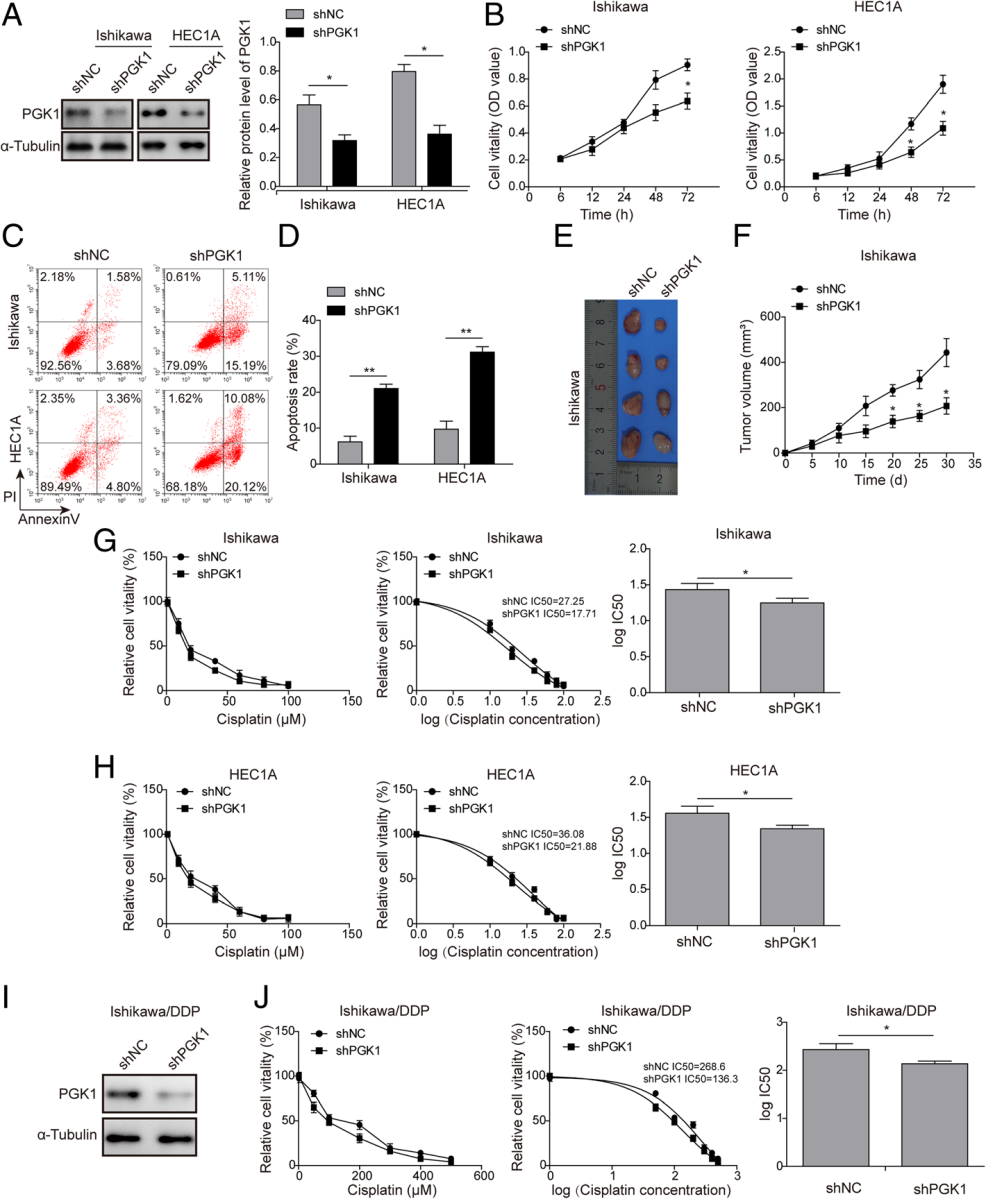
PGK1 knockdown inhibits proliferation and enhances cisplatin sensitivity in endometrial cancer cell lines. **a**. Western blot was performed to confirm knockdown of PGK1 in endometrial cancer cell lines (Ishikawa and HEC1A). α-tubulin was used as a loading control. **b**. MTT assay was used to evaluate the effects of PGK1 knockdown on cell viability in endometrial cancer cell lines (Ishikawa and HEC1A). **c**-**d**. Flow cytometry was used to assess the effect of PGK1 knockdown on apoptosis of endometrial cancer cells (Ishikawa and HEC1A). **e**-**f**. Effect of PGK1 knockdown on tumor growth in a Ishikawa cell-induced xenograft endometrial cancer model. **g**. MTT assay was used to evaluate the effect of PGK1 knockdown on the sensitivity of Ishikawa endometrial cancer cell line to cisplatin and the IC_50_ value was calculated using GraphPad Prism 6. **h**. MTT assay was used to test the effect of PGK1 knockdown on the sensitivity of HEC1A endometrial cancer cell line to cisplatin and the IC_50_ value was calculated using GraphPad Prism 6. **i**. Western blot was performed to confirm knockdown of PGK1 in cisplatin-resistant endometrial cancer cell line (Ishikawa/DDP). **j**. MTT assay was used to evaluate the effect of PGK1 knockdown on the sensitivity of Ishikawa/DDP cell line to cisplatin and the IC_50_ value was calculated using GraphPad Prism 6. Data were shown as mean ± SD based on three independent experiments. *, *P* < 0.05, **, *P* < 0.01 compared with shNC group

We next tested the effects of PGK1 on tumor cell viability in two endometrial cancer cell lines (Ishikawa and HEC1A). Knockdown of PGK1 expression was achieved by transfection with shRNA (shPGK1). Western blot confirmed that cells transfected with shPGK1 had lower expression levels of PGK1, compared with cells transfected with scrambled negative control shRNA (Fig. 2a). Cell viability was assessed by the MTT assay, and cells with PGK1 knockdown exhibited reduced cell viability in comparison with the negative control groups (shNC) (Fig. 2b). Significant difference in cell viability between the shPGK1 group and shNC group was observed after 48 h (data points at 48 h and 72 h, Fig. 2b). Next, flow cytometry was performed to assess the effect of PGK1 on cell apoptosis (Fig. 2c-d). Knockdown of PGK1 was found to promote cell apoptosis in both Ishikawa and HEC1A cell lines. To further verify the effects of PGK1 on tumor growth in vivo, we constructed a nude mouse model of endometrial cancer via injection of Ishikawa cells, and found that knockdown of PGK1 expression drastically slowed tumor growth and reduced tumor size (Fig. 2e-f). The MTT assay was further utilized to evaluate the effects of PGK1 knockdown on the sensitivity to cisplatin treatment (Fig. 2g for Ishikawa cell line, Fig. 2h for HEC1A cell line). Cell viability was measured after treatment with cisplatin for 24 h (1, 10, 20, 40, 60, 80 and 100 μM), and PGK1 knockdown was found to enhance sensitivity to cisplatin and result in lower IC_50_ values. This finding was further validated in cisplatin-resistant endometrial cell line Ishikawa/DDP. Consistently, knockdown of PGK1 was confirmed by Western blot (Fig. 2i), and was shown to increase the sensitivity of Ishikawa/DDP cells to cisplatin treatment (Fig. 2j).

### PGK1 knockdown reduces the expression of DNA repair-related proteins, methylation-related enzymes, and total cellular methylation level

**Fig. 3:**
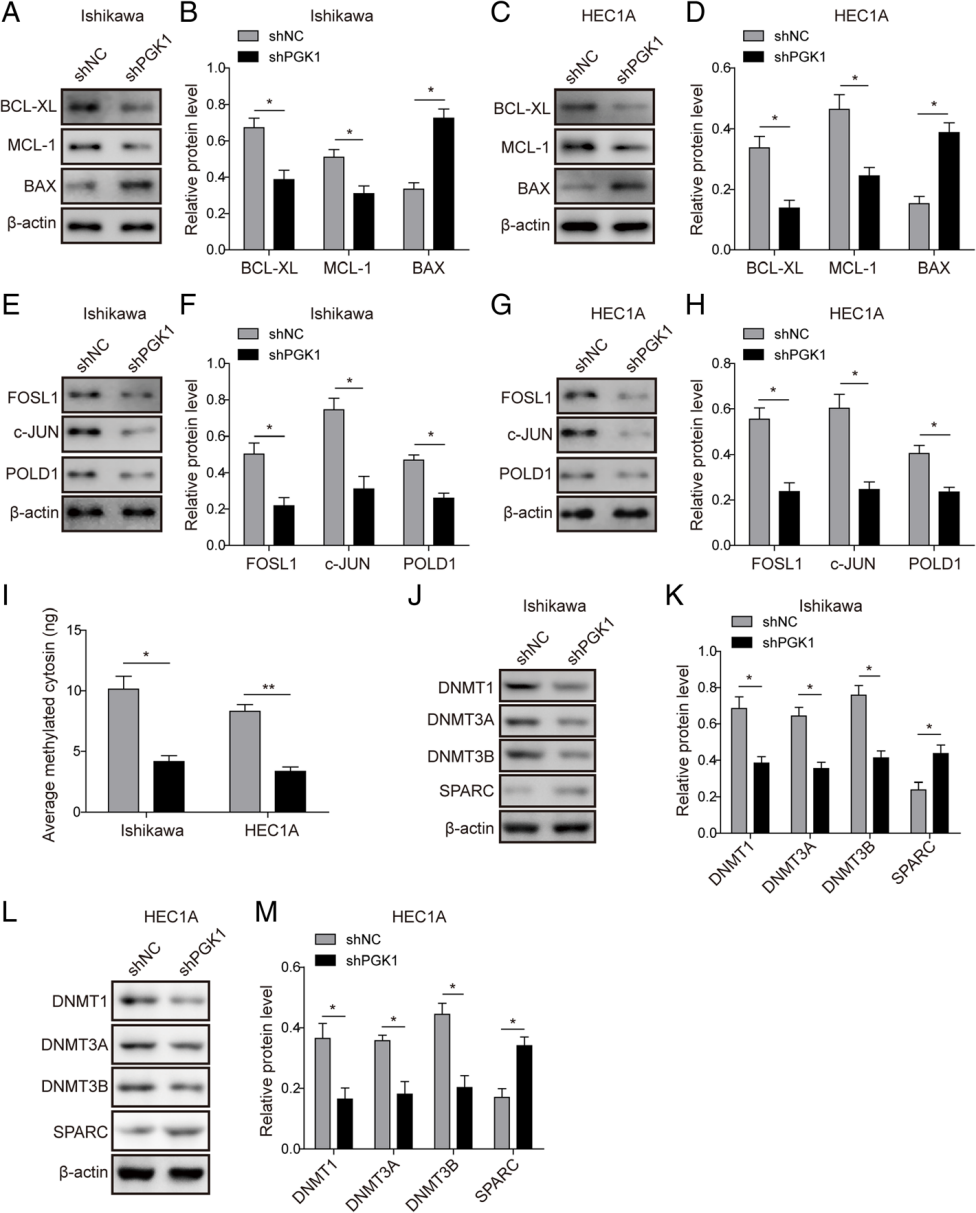
PGK1 knockdown reduces the expression of DNA repair-related proteins, methylation-related enzymes, and total cellular methylation level. **a**. Western blot was used to detect the expression of pro-survival proteins (Bcl-xL, Mcl-1) and pro-apoptotic protein (Bax) in Ishikawa endometrial cancer cell line. β-actin was used as a loading control. **b**. Semi-quantitative analysis of protein level in A. **c**. Western blot was used to detect the expression of pro-survival proteins (Bcl-xL, Mcl-1) and pro-apoptotic protein (Bax) in HEC1A endometrial cancer cell line. β-actin was used as a loading control. **d**. Semi-quantitative analysis of protein level in C. **e**. Western blot was used to detect the expression of DNA repair-related proteins c-JUN, FOSL1, and POLD1 in Ishikawa endometrial cancer cell line. β-actin was used as a loading control. **f**. Semi-quantitative analysis of protein level in A. **g**. Western blot was used to detect the expression of DNA repair-related proteins c-JUN, FOSL1, and POLD1 in HEC1A endometrial cancer cell line. β-actin was used as a loading control. **h**. Semi-quantitative analysis of protein level in C. **i**. Western blot was performed to determine the effect of PGK1 knockdown on the total intracellular methylation levels (amount of average methylated cytosine). **j**. Western blot was used to examine the effect of PGK1 knockdown on the expression of DNA methyltransferases (DNMT1, DNMT3A, and DNMT3B) and SPARC in Ishikawa cancer cell line. β-actin was used as a loading control. **k**. Semi-quantitative analysis of protein level in F. **l**. Western blot was used to examine the effect of PGK1 knockdown on the expression of DNA methyltransferases (DNMT1, DNMT3A, and DNMT3B) and SPARC in HEC1A endometrial cancer cell line. β-actin was used as a loading control. **m**. Semi-quantitative analysis of protein level in H. Data were shown as mean ± SD based on three independent experiments. *, *P* < 0.05, **, *P* < 0.01 compared with shNC group

Next, we assessed the effect of PGK1 on the expression of cell survival and apoptosis-related proteins by Western blot (Fig. 3a-b for Ishikawa cell line, Fig. 3c-d for HEC1A cell line). Compared with the negative control, PGK1 knockdown decreased the expression of pro-survival proteins (Bcl-xL and Mcl-1), while increased the expression of pro-apoptotic protein (Bax). We further defined the role of PGK1 in endometrial cancer by assessing the impact of its knockdown on DNA repair and methylation. We found by Western blot that the expression of DNA repair-related proteins (c-JUN, FOSL1, and POLD1) was significantly down-regulated after knockdown of PGK1 (Fig. 3e-f for Ishikawa cell line, Fig. 3g-h for HEC1A cell line). We next analyzed the total cellular methylation levels by quantification of methylated cytosin, and found knockdown of PGK1 resulted in at least two fold decrease in total cellular methylation in both Ishikawa and HEC1A cell lines (Fig. 3i). Meanwhile, expression of DNA methyltransferases (DNMT1, DNMT3A, and DNMT3B) was found to be down-regulated after PGK1 knockdown (Fig. 3j-k for Ishikawa cell line, Fig. 3l-m for HEC1A cell line). In addition, we observed up-regulation of secreted protein acidic and rich in cysteine (SPARC), which has been reported to be inactivated by promoter methylation in cancer (Yang et al., 2007; Yusuf et al., 2014).

### PGK1 knockdown inhibits phosphorylation of ERK, JNK, and AKT pathway

**Fig. 4:**
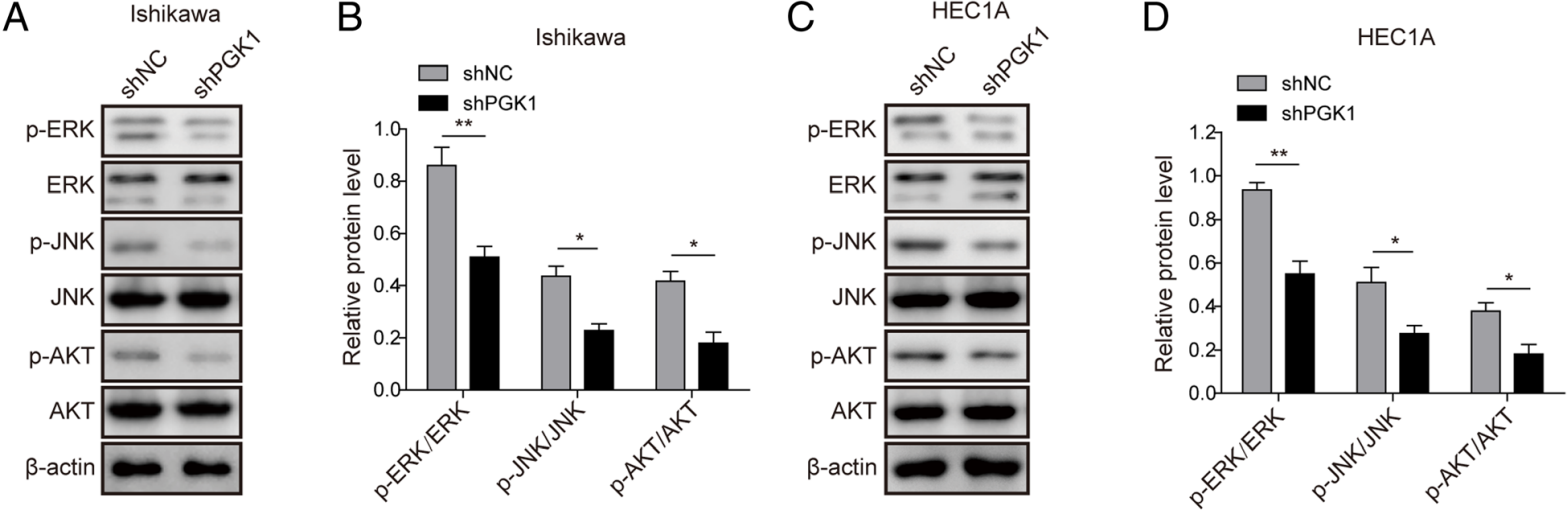
PGK1 knockdown inhibits phosphorylation of JNK, ERK, and AKT pathway. **a**. Western blot was performed to evaluate the effects of PGK1 knockdown on phosphorylation levels of JNK, ERK, and AKT in Ishikawa cancer cell line. β-actin was used as a loading control. **b**. Semi-quantitative analysis of protein level in A. **c**. Western blot was performed to evaluate the effects of PGK1 knockdown on phosphorylation levels of JNK, ERK, and AKT pathway in HEC1A cancer cell line. β-actin was used as a loading control. **d**. Semi-quantitative analysis of protein level in C. Data were shown as mean ± SD based on three independent experiments. *, *P* < 0.05, **, *P* < 0.01 compared with shNC group

We further investigated the downstream molecular mechanism by which PGK1 regulates cancer cell proliferation. AKT and mitogen-activated protein kinases (MAPKs) including ERK and JNK have been found to be dysregulated in multiple tumor types (Fang & Richardson, 2005; Sun et al., 2018). Western blot validated that knockdown of PGK1 significantly reduced the phosphorylation levels of ERK, JNK and AKT (Fig. 4a-b for Ishikawa cell line, Fig. 4c-d for HEC1A cell line). The above result indicates that knockdown of PGK1 induced decreased activation of the ERK, JNK, and AKT pathway.

### PGK1 interacts directly with HSP90 and modulates the ATPase activity of HSP90

**Fig. 5:**
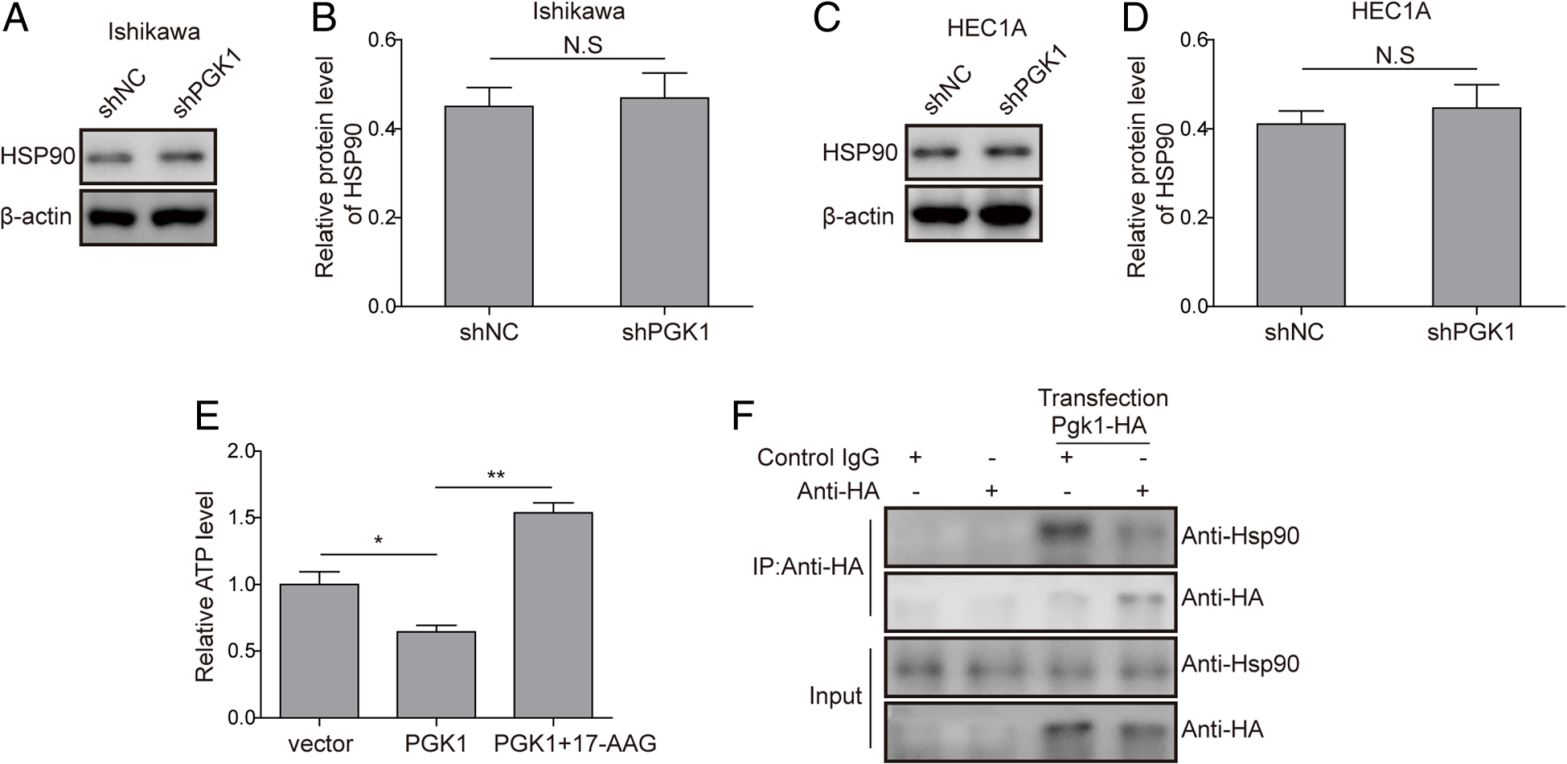
PGK1 interacts directly with HSP90 and modulates the ATPase activity of HSP90**. a**. Western blot was performed to assess the effects of PGK1 knockdown on expression levels of HSP90 in Ishikawa cancer cell line. β-actin was used as a loading control. **b**. Semi-quantitative analysis of protein level in A. **c**. Western blot was performed to assess the effects of PGK1 knockdown on expression levels of HSP90 in HEC1A cancer cell line. β-actin was used as a loading control. **d**. Semi-quantitative analysis of protein level in C. **e**. Effect of PGK1 overexpression and HSP90 inhibitor 17-AAG on cellular ATP level. Vector, empty vector; PGK1, vector for PGK1 overexpression; PGK1 + 17-AAG, vector for PGK1 overexpression supplemented with 17-AAG. **f**. Co-immunoprecipitation was used to detect binding between PGK1 and HSP90. Hemagglutinin (HA)-tagged PGK1 (PGK1-HA) was overexpressed in Ishikawa cells. Data were shown as mean ± SD based on three independent experiments. *, *P* < 0.05, **, *P* < 0.01 compared with shNC, empty vector or PGK1 overexpressed (PGK1) group respectively

To validate the regulatory relationship between PGK1 and HSP90, we first showed by Western blot that knockdown of PGK1 did not affect the expression level of HSP90 (Fig. 5a-b for Ishikawa cell line, Fig. 5c-d for HEC1A cell line). Since the function of HSP90 is dependent on the ATPase activity (Pearl, 2016), we tested if the ATPase activity is regulated by PGK1 levels by measuring cellular ATP levels. The results indicated that overexpression of PGK1 down-regulated ATP levels, whereas if PGK1 overexpression is accompanied by addition of HSP90 ATPase inhibitor 17-AAG (Dimopoulos et al., 2011), the decrease in ATP level was abrogated (Fig. 5e). Furthermore, we investigated that if direct interactions exist between PGK1 and HSP90 by overexpressing hemagglutinin (HA)-tagged PGK1 in Ishikawa cells, followed by immunoprecipitation (IP) with anti-HA antibody. We found that HSP90 can be co-precipitated with PGK1, and confirmed that PGK1 regulates HSP90 through direct binding (Fig. 5f).

### PGK1 facilitates chemoresistance to cisplatin through HSP90/ERK pathway

**Fig. 6:**
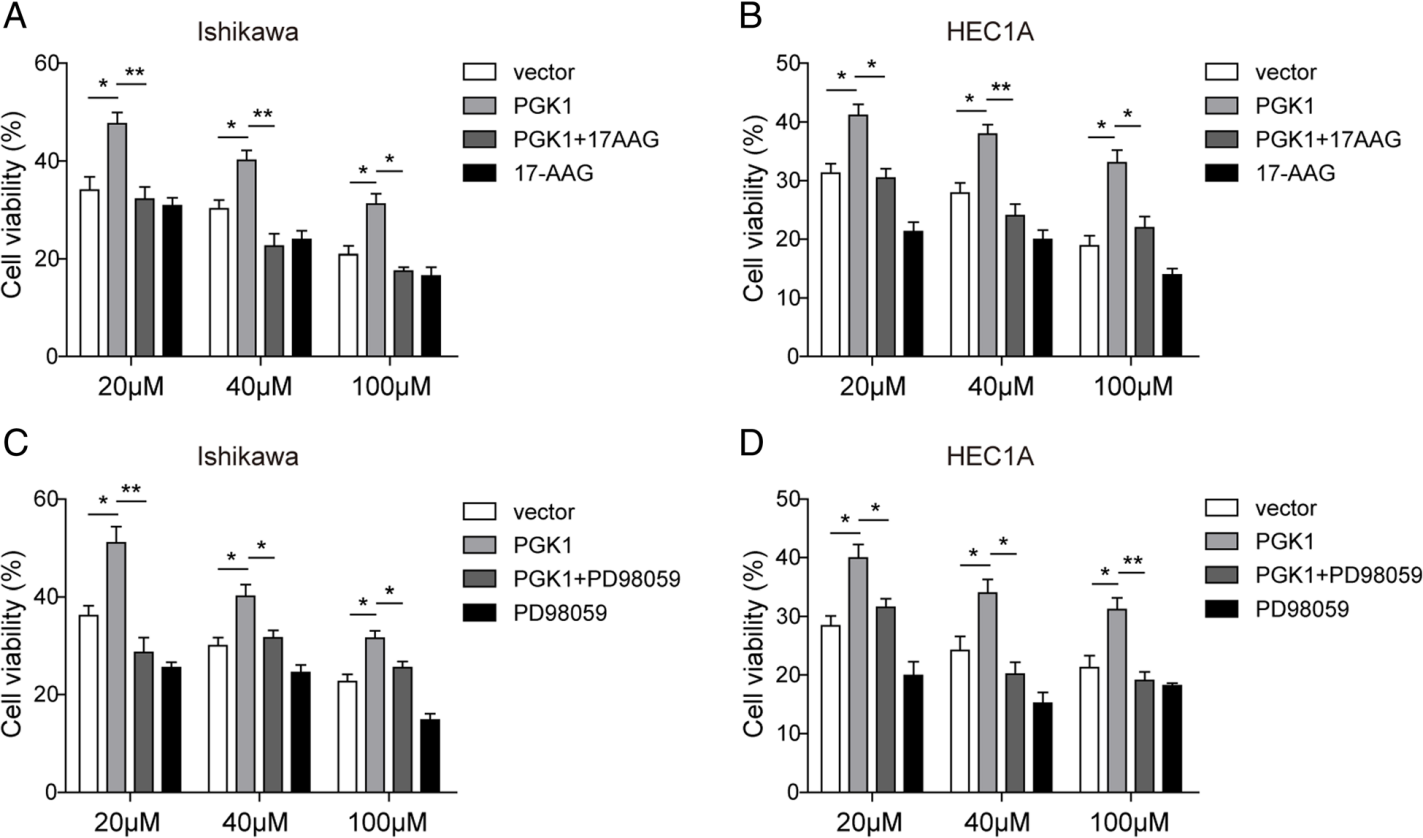
PGK1 facilitates chemoresistance to cisplatin through HSP90/ERK pathway. **a**-**b**. MTT assay was used to evaluate the effects of PGK1 overexpression or HSP90 ATPase inhibitor 17-AAG on chemoresistance to cisplatin in Ishikawa (**a**) and HEC1A (**b**) cell lines. Vector, empty vector; PGK1, plasmid for PGK1 overexpression; PGK1 + 17-AAG, plasmid for PGK1 overexpression supplemented with 17-AAG. **c**-**d**. MTT assay was used to evaluate the effects of PGK1 overexpression or ERK pathway inhibitor PD98059 on chemoresistance to cisplatin in Ishikawa (**c**) and HEC1A (D) cell lines. Vector, empty vector; PGK1, plasmid for PGK1 overexpression; PGK1+ PD98059, plasmid for PGK1 overexpression supplemented with PD98059. Data were shown as mean ± SD based on three independent experiments. *, *P* < 0.05, **, *P* < 0.01 compared with empty vector group or PGK1 overexpressed group respectively

We next investigate whether the promotive effects of PGK1 on the chemoresistance in endometrial cancer cell lines is dependent on HSP90 and the downstream ERK pathway. MTT assay was employed to determine cell viability after exposure to cisplatin at 20, 40, or 60 μM for 72 h. We found overexpression of PGK1 resulted in increased cell viability in comparison with negative control using an empty vector (Fig. 6a-d). However, if overexpression of PGK1 was supplemented with HSP90 ATPase inhibitor 17-AAG (Fig. 6a-b) or ERK pathway inhibitor PD98059 (Fig. 6c-d) (Alessi et al., 1995), the sensitivity of the endometrial cancer cell lines to cisplatin was restored. Treatment with 17-AAG or PD98059 alone also enhanced the inhibitory effect of cisplatin on cell viability (Fig. 6a-d). Therefore, we propose that PGK1 regulates chemoresistance to cisplatin at least partly through the HSP90/ERK pathway.

### HSP90 inhibitor 17-AAG exhibits similar effects as knockdown of PGK1

**Fig. 7:**
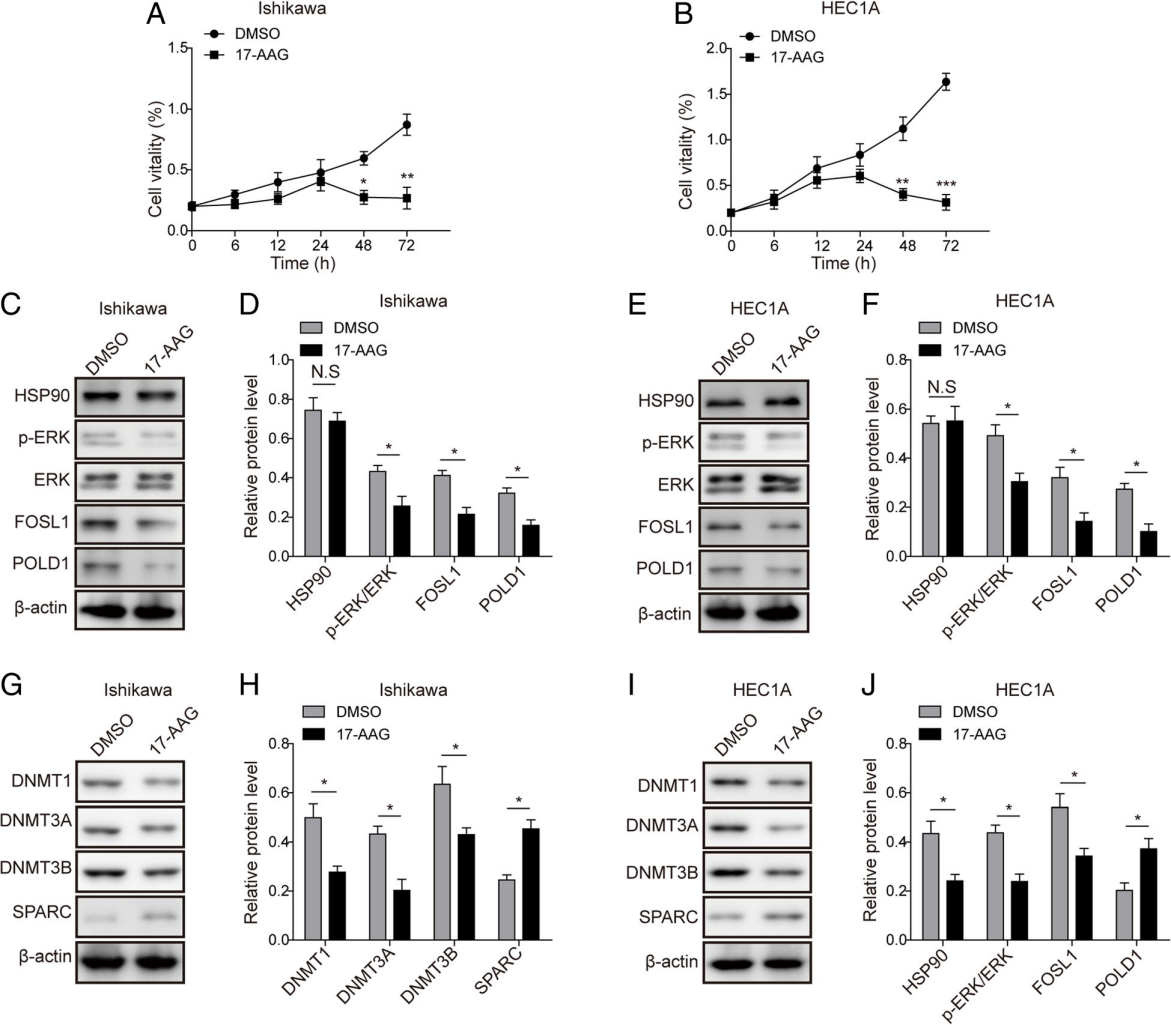
HSP90 inhibitor 17-AAG exhibits similar effects as knockdown of PGK1. **a**-**b**. MTT assay was used to evaluate the effects of HSP90 inhibitor 17-AAG on cell viability in endometrial cell lines Ishikawa (**a**) and HEC1A (**b**). DMSO was used as negative control. **c**. Western blot was used to detect the expression of DNA repair-related proteins c-JUN, FOSL1, and POLD1 in the presence of HSP90 inhibitor 17-AAG in Ishikawa cancer cell line. β-actin was used as a loading control. **d**. Semi-quantitative analysis of protein level in C. **e**. Western blot was used to detect the expression of DNA repair-related proteins c-JUN, FOSL1, and POLD1 in the presence of HSP90 inhibitor 17-AAG in HEC1A cancer cell line. β-actin was used as a loading control. **f**. Semi-quantitative analysis of protein level in E. **g**. Western blot was performed to examine the effect of HSP90 inhibitor 17-AAG on the expression of DNA methyltransferases (DNMT1, DNMT3A, and DNMT3B) and SPARC in Ishikawa cancer cell line. β-actin was used as a loading control. **h**. Semi-quantitative analysis of protein level in G. **i**. Western blot was performed to examine the effect of HSP90 inhibitor 17-AAG on the expression of DNA methyltransferases (DNMT1, DNMT3A, and DNMT3B) and SPARC in HEC1A cancer cell line. β-actin was used as a loading control. **j**. Semi-quantitative analysis of protein level in I. Data were shown as mean ± SD based on three independent experiments. *, *P* < 0.05, **, *P* < 0.01 compared with DMSO group

To further support the hypothesis that PGK1 functions by modulating HSP90, we validated that HSP90 inhibitor 17-AAG mimicked the phenotype of PGK1 knockdown on chemoresistance, DNA-repair, and DNA methylation (Fig. 7). In the presence of cisplatin, MTT assay showed that the supplement of 17-AAG resulted in drastic decrease in cell vitality after 48 h compared with the negative control using DMSO, indicating elevated sensitivity to cisplatin (Fig. 7a for Ishikawa cell line, Fig. 7b for HEC1A cell line). This finding is consistent with the effects of PGK1 knockdown on cisplatin sensitivity (Fig. 2b). Western blot showed that 17-AAG did not affect the expression level of HSP90, but down-regulated the expression of DNA repair-related proteins (FOSL1 and POLD1) and decreased the phosphorylation level of ERK (Fig. 7c-d for Ishikawa cell line, Fig. 7e-f for HEC1A cell line). Moreover, 17-AAG was found to down-regulate the expression of DNA methyltransferases (DNMT1, DNMT3A, and DNMT3B), and up-regulate the expression of SPARC (Fig. 7g-h for Ishikawa cell line, Fig. 7i-j for HEC1A cell line). The above expression profile of DNA repair and DNA methylation-related proteins was in accordance with the inhibitory effects induced by PGK1 knockdown (Figs. 3 and 4).

## Discussion

In brief, the present study showed that PGK1 knockdown increased the sensitivity of endometrial cancer cells to cisplatin by down-regulating the expression of DNA repair and methylation-related genes in a HSP90-dependent manner.

Phosphoglycerate kinase 1 (PGK1) is a glycolytic enzyme that has recently been shown to be related to the poor prognosis of various types of cancers (Ahmad et al., 2013; Ai et al., 2011; Yan et al., 2012; Zhang et al., 2005; Zieker et al., 2010). PGK1 has been revealed to promote tumor proliferation and metastasis in various types of cancers, including astrocytoma (Yan et al., 2012), gastric cancer (Ahmad et al., 2013) and colon cancer (Zieker et al., 2010). In addition, overexpression of PGK1 has been indicated to be associated with multidrug resistance phenotypes (Duan et al., 2002), but the underlying mechanism remains poorly understood. Moreover, PGK-1 has been demonstrated to be overexpressed in endometrial carcinoma and to be associated with poor prognosis, while its specific function in endometrial cancer is unknown (Guo et al., 2018). This report, for the first time, unveiled the mechanism by which PGK1 mediates chemoresistance in endometrial cancer.

In the present study, we provide evidence that PGK1 regulated chemoresistance by modulating the ATPase activity of HSP90, consistent with the previous finding that PGK1 activates HSP90 ATPase in response to cellular stress (Chen et al., 2015). Methylation of DNA is catalyzed by the DNA methyltransferase (DNMT) family of enzymes using S-adenosyl methionine (SAM) as the methyl group donor. Epigenetic modifications have been reported to play key roles in the proliferation and progression of tumors (Yoon et al., 2010; Yoshikawa et al., 2001), and dysregulated DNA hypermethylation have been implicated in the resistance to multiple chemotherapeutics (Strathdee et al., 2005; Tamura, 2009). DNMTs have been identified as client proteins of HSP90, and inhibition of HSP90 expression results in downregulation of DNMT1, DNMT3A and DNMT3A expression (Nagaraju et al., 2017). In present study, we showed that PGK1 knockdown and HSP90 inhibitor 17-AAG resulted in down-regulation of DNMTs expression. Therefore, we propose that up-regulation of DNMTs expression may be involved in PGK1-mediated chemoresistance in endometrial carcinoma.

In addition to DNA methylation-related proteins, we revealed that PGK1 influenced the expression of pro-survival, pro-apoptotic and DNA repair-related proteins. There are several well-studied DNA repair-related proteins in cancer research. For instance, c-Jun has been shown to be overexpressed in diverse cancer types (Smith et al., 1999; Szabo et al., 1996), FOSL1 has been implicated as a regulator of cell proliferation and differentiation (Matsui et al., 1990), and mutations of polymerase delta 1 (POLD1), which functions in proofreading of DNA replication and replication-linked DNA repair (Prindle & Loeb, 2012), have been associated with a variety of cancer types (Rayner et al., 2016). Recently, the above proteins c-JUN, FOSL1, and POLD1 have been shown to mediate chemoresistance to cisplatin in esophageal squamous cell carcinoma regulated by HSP90/ERK signaling (Sun et al., 2018). Herein, we demonstrated that knockdown of PGK1 inhibited the expression of c-JUN, FOSL1, and POLD1, indicating that PGK1 regulates chemoresistance in endometrial carcinoma through upregulation of DNA repair-related proteins. Taken together, we demonstrated that DNA repair and methylation are involved in PGK1/HSP90-mediated chemoresistance.

HSP90 is known to regulate downstream pro-tumor signaling pathways through its ATPase activity (Neckers, 2007). In present work, we for the first time verified that PGK1 directly binds HSP90 and that overexpression of PGK1 down-regulates cellular ATP level in endometrial cancer cell lines, which is consistent with previous finding that HSP90 ATPase is activated by terazosin through PGK1 under cellular stress (Chen et al., 2015). We showed that the effect of PGK1 overexpression is compromised by HSP90 inhibitor 17-AAG, suggesting that the function of PGK1 on chemoresistance is at least partly dependent on HSP90. At the same time, we would like to point out that PGK1 may also affect endometrial cancer chemoresistance in HSP90-independent manners, including by regulating autophagy, glycolysis, or the tricarboxylic acid (TCA) cycle (Ariosa & Klionsky, 2017; Li et al., 2016; Qian et al., 2017a; Qian et al., 2017b). It was shown in a recent report that PGK1, as a key kinase in glycolysis and the TCA cycle, regulates PDHK1 T338 phosphorylation and promotes tumorigenesis in glioblastoma (Li et al., 2016). It remains to be investigated whether PGK1 could regulate chemoresistance of endometrial cancer by affecting cellular metabolism, and this possible underlying mechanism should be further investigated in future work. 17-N-allylamino-17-demethoxygeldanamycin (17-AAG), also known as tanespimycin, is a derivative of geldanamycin that inhibits the ATPase activity of HSP90 (Dimopoulos et al., 2011). Meanwhile, the present work revealed, for the first time, that 17-AAG alone enhances the inhibitory effect of cisplatin on cell viability in endometrial cancer cell lines.

## Conclusions

Taken together, we elucidated in this work a regulatory axis consisting of PGK1, HSP90, ERK pathway, DNA methylation and DNA repair-related proteins to regulate cisplatin chemoresistance in endometrial carcinoma. We defined that PGK1 facilitates chemoresistance to cisplatin by activating the HSP90/ERK pathway-mediated DNA methylation and DNA repair. This work lays the foundation for additional in-depth mechanistic studies, and for the development of novel therapeutics for endometrial carcinoma.

## Abbreviations

17-AAG: 17-N-allylamino-17-demethoxygeldanamycin
DMEM: Eagle’s minimal essential medium
DNMT1: DNA methyltransferase
ERK: Extracellular signal-regulated kinase
FBS: Fetal bovine serum
FRA1: Fos-related antigen 1
HA: Hemagglutinin
HSP90: Heat shock protein 90
JNK: Jun N-terminal kinases
MAPK: Mitogen-activated protein kinase
PBS: Phosphate-buffered saline
PGK1: Phosphoglycerate kinase 1
POLD1: Polymerase delta 1
PVDF: Polyvinylidene difluoride
qPCR: quantitative polymerase chain reaction
RIP3: interacting protein kinase 3
SAM: S-adenosyl methionine
shRNA: Small hairpin ribonucleic acid
SPARC: Secreted protein acidic and rich in cysteine
